# A focus on microporous perovskites: new tricks for an old dog

**DOI:** 10.1039/d4sc90244k

**Published:** 2025-01-06

**Authors:** Miriam Segundo-Osorio, A. Paulina Gómora-Figueroa, Diego Solis-Ibarra

**Affiliations:** a Laboratorio de Fisicoquímica y Reactividad de Superficies (LaFReS), Instituto de Investigaciones en Materiales, Universidad Nacional Autónoma de México Circuito Exterior s/n, CU, Coyoacán 04510 Ciudad de México Mexico diego.solis@unam.mx; b División de Ingeniería en Ciencias de la Tierra, Facultad de Ingeniería, Universidad Nacional Autónoma de México, Ciudad Universitaria Circuito Exterior 04510 Ciudad de México Mexico

## Abstract

Hybrid organic–inorganic perovskites (HOIPs) are widely studied for their potential in optoelectronic devices due to their unique semiconductor features. Porous HOIPs are extremely rare, with (APOSS)[CuCl_4_]_4_ being one of the very few examples, featuring 12 Å pores within its lattice. Reed and coworkers (C. W. Dalton, P. M. Gannon, W. Kaminsky and D. A. Reed, *Chem. Sci.*, 2025, DOI: https://doi.org/10.1039/D4SC04378B) have recently shed light on the structure of this interesting material and demonstrated that these pores can incorporate large electroactive molecules such as ferrocene (Fc) and tetracyanoethylene (TCNE). Further, they showed that the ability to incorporate molecules within the pores also enables the synthesis of new crystalline phases and unlocks numerous applications, including gas sensing and photocatalysis, among others.

## Abstract 2

Microporous hybrid perovskites merge porosity with semiconducting properties, offering tunable properties *via* guest molecule incorporation, and unlocking new phases, reactivity, and potential new applications in sensing, photocatalysis, and beyond.

## Graphical abstract

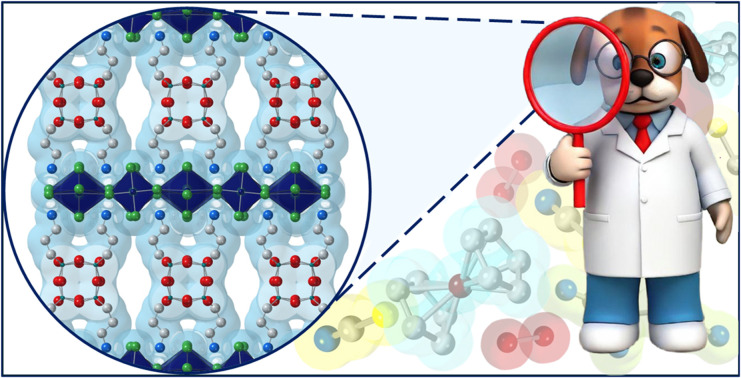

Hybrid organic–inorganic perovskites (HOIPs) have been attracting considerable interest due to their versatile application potential in light emitting diodes (LEDs),^1^ solar cells,^2–4^ photocatalysis^5^ and beyond.^6–10^ Most of these applications are possible thanks to their extended, two- or three-dimensional network of overlapping orbitals, which collectively form the electronic structure characteristic of a semiconductor. As an intense orbital overlap is needed, close packing is usually a prerequisite, making porous semiconductors exceptionally rare. In fact, there is only one report of a permanently porous HOIP: (APOSS)[MCl_4_]_4_ (where APOSS is an octylammonium polyhedral oligomeric silsesquioxane and M = Cu, Mn or Pb). Originally reported by Kataoka and collaborators,^11^ (APOSS)[MCl_4_]_4_ showed an impressive surface area of up to 205 m^2^ g^−1^. However, Kataoka and colleagues were unable to obtain a reliable crystal structure or to show if this porosity could accommodate molecules larger than dinitrogen.

In a recently published article, Reed and coworkers,^12^ have been able to obtain a high-quality single-crystal X-ray structure of (APOSS)[CuCl_4_]_4_, revealing a structure with a unique arrangement of siloxane cores, with adjacent rows of POSS groups that shift to create 12 Å pores (Fig. 1).

**Fig. 1:**
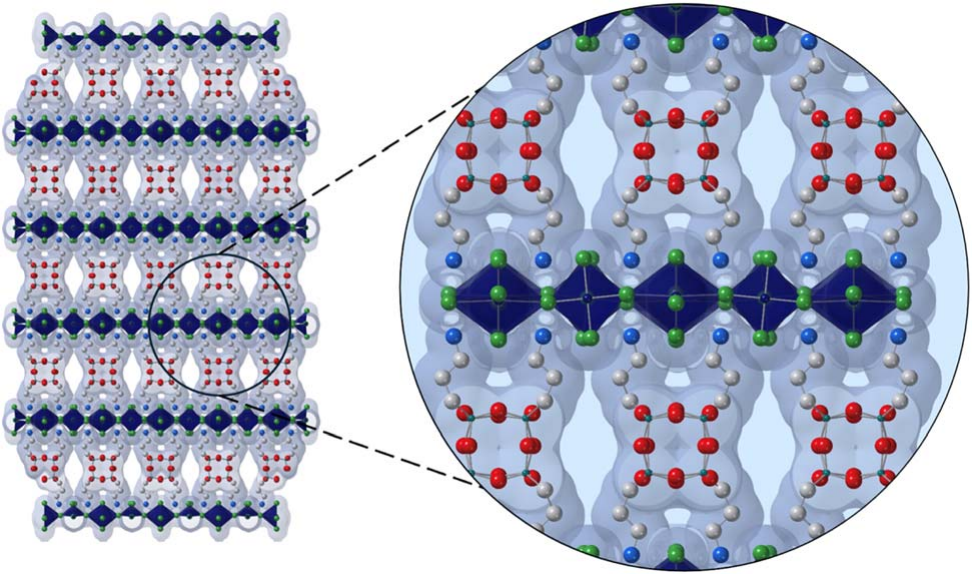
Structure of (APOSS)[CuCl_4_]_4_. Dark blue, green, white, light blue, red, and teal spheres represent Cu, Cl, C, N, O, and Si, respectively. H atoms and disordered parts are omitted for clarity.

Beyond characterizing the structure, the authors demonstrated that electroactive molecules like ferrocene (Fc) and tetracyanoethylene (TCNE) can be introduced by simply soaking the material in a solution of either molecule or by exposing it to Fc vapors. Remarkably, Fc adsorption turns the originally yellow crystals a deeper yellow, a color change that persists after washing, suggesting successful incorporation of Fc into the perovskite lattice. The presence of TCNE and Fc was confirmed *via* several techniques, indicating that more than one Fc molecule per pore can be retained within the material. Additionally, Reed and coauthors found that the loading amount is tunable based on the duration of Fc exposure, with significant structural adjustments occurring when larger quantities are introduced. Experimental analyses showed that TCNE molecules undergo a similar loading mechanism within the porous perovskite lattice (Fig. 2).

The ease of incorporating Fc and TCNE is particularly exciting for two reasons: first, it confirms the accessibility of the pores and the ability of these materials to adsorb and desorb relatively large molecules; and second, it opens the door to numerous new research avenues. For example, the authors showed that the porosity of (APOSS)[CuCl_4_]_4_, gives unprecedented access to the inorganic layers of these HOIPs, thereby allowing for previously unavailable reactivity. Specifically, the authors substituted chloride with bromide, enabling the formation of a new, previously unobserved, crystalline phase that retains the porosity of the original material. Similarly, the authors also show that pseudohalides, such as thiocyanate, can replace terminal halides in certain perovskites. Once again, this introduces a previously unknown structure enabled by porosity: (APOSS)[CuCl_4−*x*_(SCN)_*x*_]_4_ where SCN has replaced the terminal chlorides while preserving the HOIP structure and the lattice porosity (Fig. 2).

**Fig. 2:**
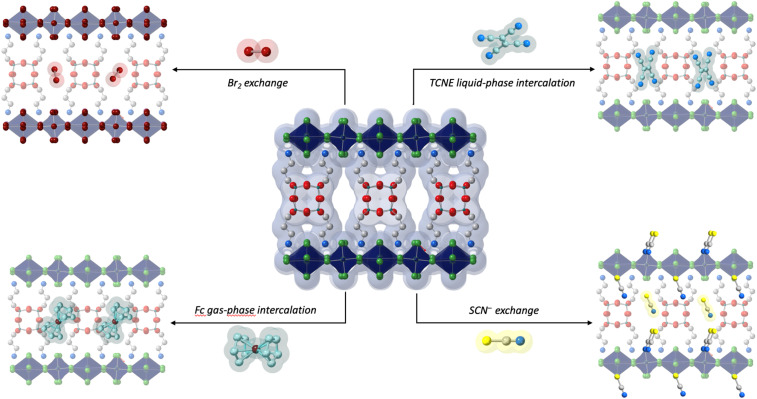
Schematic representation of the adsorptive and reactive properties of (APOSS)[CuCl_4_]_4_. Dark blue, green, white, light blue, red, and teal spheres represent Cu, Cl, C, N, O, and Si, respectively. H atoms and disordered parts are omitted for clarity.

Besides unusual reactivity and the new materials that stem from it, microporous HOIPs could enable several other avenues of research and applications. For instance, the incorporation of controlled amounts of electroactive guest molecules with appropriate energetic levels could be used to modulate the material's optical and electronic properties. Moreover, the presence of a semiconducting (and often emissive) inorganic layer opens up potential uses in small molecule photocatalysis or small molecule sensors.^13–15^

In summary, microporous HOIPs represent a promising frontier in materials science, merging the unique reactivity and functionality of porous structures with the electronic advantages of hybrid perovskites. By enabling tuneable optical and electronic properties through guest molecule incorporation, these materials hold vast potential for applications extending beyond conventional uses. As research continues to explore, understand and apply microporous HOIPs, the possibilities are vast, with promise in fields such as photocatalysis, sensing and beyond.

## Author contributions

M. S.-O., A. P. G.-F. and D. S.-I. wrote the manuscript.

## Conflicts of interest

There are no conflicts to declare.
